# Strategies to Determine and Mitigate Pesticide Residues in Food

**DOI:** 10.3390/molecules31010063

**Published:** 2025-12-24

**Authors:** Ana Rita Oliveira, Sílvia Cruz Barros, Duarte Torres, Ana Sanches Silva

**Affiliations:** 1 University of Coimbra, Faculty of Pharmacy, Polo III, Azinhaga de Stª Comba, 3000-548 Coimbra, Portugal; anaritaoliver.03@gmail.com; 2 National Institute for Agricultural and Veterinary Research (INIAV), I.P., Rua dos Lagidos, Lugar da Madalena, Vairão, 4485-655 Vila do Conde, Portugal; silvia.barros@iniav.pt; 3 Faculty of Nutrition and Food Sciences, University of Porto, 4200-393 Porto, Portugal; dupamato@fcna.up.pt; 4 EPIUnit-Institute of Public Health, University of Porto, 4200-450 Porto, Portugal; 5 Laboratory for Integrative and Translational Research in Population Health (ITR), 4200-450 Porto, Portugal; 6 Center for Study in Animal Science (CECA), ICETA, University of Porto, 4050-453 Porto, Portugal; 7 Associate Laboratory for Animal and Veterinary Sciences (Al4AnimalS), 1300-477 Lisbon, Portugal

**Keywords:** pesticide mitigation, pesticide residues, household processing, artificial intelligence, pesticide classification

## Abstract

This review provides a comprehensive overview of strategies to mitigate pesticide residues in food, examining both household and industrial processing techniques alongside the emerging role of Artificial Intelligence (AI). Simple household methods, such as washing, peeling, and thermal processing (e.g., boiling, frying), are effective. For instance, washing with running water achieves a reduction of up to 77% in residue for some vegetables. Additionally, processes like jam-making or frying can significantly reduce specific residues. Industrially, advanced methods such as ozonated water washing, ultrasonification, and cold plasma are employed for high efficiency while preserving food quality. Critically, AI is emerging as a powerful, indirect tool through predictive modelling, AI-assisted sorting/screening, and consumer guidance, enhancing precision agriculture and regulatory analytics. The review paper concludes that a combination of these diverse methods is essential for minimizing pesticide exposure and ensuring a safer food supply.

## 1. Introduction

The world population is in constant growth, and with it, there is a corresponding need to increase food production at a sufficient rate, which in turn increases the use of agricultural pesticides [1].

Pesticides play a particularly significant role in modern food production, serving as a means to protect crops from pests, diseases, and weeds. They also ensure global food safety by maximizing crop yields, allowing farmers to produce more food on less land. Around one-third of agricultural products rely on pesticides, and without them, productivity would be severely compromised due to improper pest control [2].

One alternative to the use of pesticides in agriculture is integrated pest management (IPM), which involves carefully considering all available methods for protecting plants and implementing them in crops. Some of the measures could be: (i) crop rotation; (ii) adequate cultivation techniques; (iii) use of balanced fertilization, liming and irrigation practices; (iv) preventing the spread of harmful organisms through proper hygiene measures; (v) monitorization of the harmful organisms; (vi) non-chemical methods instead of chemical methods, if they provide reasonable pest control [3]. There is, however, a need to implement several IPM measures in combination to achieve an effective and sustainable approach [4].

It is also important to understand the impacts of climate change, a persistent concern of our time. Understanding how climate change, a recurring issue in modern times, affects the behavior and efficacy of pesticides is equally crucial. The heat stress and changes in rainfall patterns make crops more vulnerable to pests. Insects can sense plants’ vulnerability and therefore impact crop productivity. The increases in temperature and shifts in moisture will cause insects to have a higher development rate and a shift in their geographic range, as well as an impact on winter survival. Consequently, it will be necessary to increase the use of pesticides on the affected crops. This increase in temperature, however, will also cause the volatilization of pesticides, meaning that instead of reaching their target, a percentage of pesticides will end up in the air, causing pollution and health problems [5].

The presence of pesticides in food is a growing concern in society, particularly with foods that are often eaten raw, such as fruits and vegetables. In light of these concerns, important questions emerge, such as which home food treatment methods are most effective in eliminating pesticide residues and whether certain classes of pesticides are more resistant to removal by conventional techniques.

A report from the Pesticide Action Network (PAN) Europe analyzed data from 2011 to 2021 related to pesticide residue monitoring programs in Member States [6]. Their analysis showed that in a total of 278.516 samples holding both fruits as vegetables, which met their criteria for the study, 23.256 of those held per- and polyfluoroalkyl substances (PFAS), a group of over 4700 heterogeneous compounds with amphipathic properties and stability to thermal and chemical degradation, with a wide use in industrial sectors and an increasing concern due to the multiple ways of exposure [7], meaning that on average 8.40% of the samples were contaminated. A maximum of 5 PFAS was detected in a single sample, and a total of 31 PFAS were detected across all samples.

Among EU-grown fruits, apricots, peaches, and strawberries stood out for having a higher proportion of contaminated samples—and worryingly, those numbers have been rising over the years. As for imported fruits, table grapes, bananas, and apricots were among the most frequently contaminated. Within the EU-grown vegetables, chicories, cucumbers, and peppers stood out as the most contaminated, while on the EU-imported vegetables, cucumbers, aubergines, and peppers were considered the most contaminated. Regarding pesticides, the most frequently detected in EU-grown products were fluopyram, flonicamid, and trifloxystrobin. In EU-imported products, the most frequently detected were fluopyram, bifenthrin, and trifloxystrobin [6].

Furthermore, the European Food Safety Authority (EFSA) releases annual reports assessing pesticide residue levels in the European market (Table 1). The following table summarizes the key findings from the 2021, 2022, and 2023 reports [8,9,10].

With the advance of the food industry, it became necessary to classify foods into distinct categories based on the processing they undergo before reaching supermarket shelves and, ultimately, our homes, since it is also required to evaluate the contamination level in these types of products. This way, Food Classification Systems were created, and some of them considered the Degree of Processing that food was subjected to. From the first-ever classification system issued by the National Institute of Public Health (NIPH) of Mexico in 2007 to the 2018 systems created by the Food Standards Australia New Zealand, although this one only distinguished beverages and food into processed and non-processed categories without taking into consideration the degree of processing [11].

**Figure 1:**
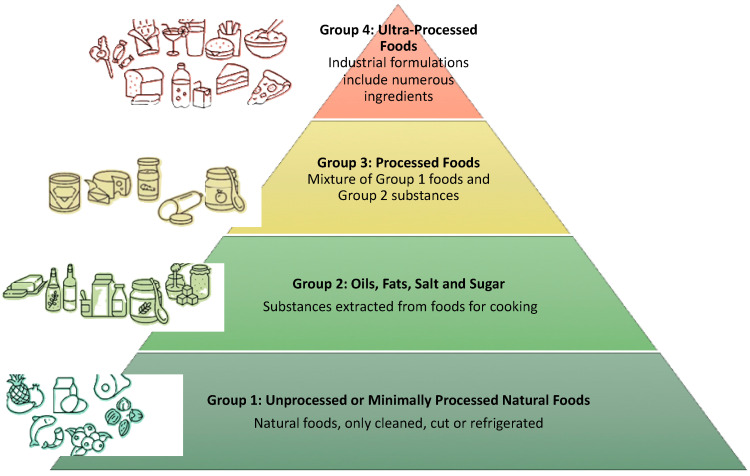
NOVA Food Classification System [13].

In 2009, researchers from the University of São Paulo in Brazil created the NOVA system (new system), which separates foods into groups based on the extent and purpose of the processing that they go through (Figure 1). The classification was updated in 2015 and is now recognized as the most applied system in scientific literature [12]. According to this classification, food is divided into four groups: unprocessed or minimally processed foods; oils, fats, salt, and sugar; processed foods; and ultra-processed foods [13]. Foods can be classified into four groups. Group 1, unprocessed or minimally processed foods, includes items obtained directly from plants or animals that undergo no alteration beyond cleaning, removal of inedible parts, or simple treatments such as freezing or pasteurization; examples are vegetables, fruits, potatoes, eggs, coffee, tea, water, nuts, and fresh or pasteurized milk [13]. Group 2, consisting of oils, fats, salt, and sugar—also known as processed culinary ingredients—comprises products extracted from natural sources through pressing, grinding, crushing, or refining, such as vegetable oils, sugar, honey, butter, and coconut fat [13]. Group 3, processed foods, are produced by adding ingredients from Group 2 to Group 1 foods to preserve them or enhance flavor, including canned fish, tomato pastes, bacon, salted or sugared nuts and seeds, fermented alcoholic beverages, and freshly made cheeses [13]. Group 4, ultra-processed foods, are industrial formulations made from food-derived substances or laboratory-synthesized ingredients, often created through techniques like extrusion, molding, or frying; this group includes ice creams, carbonated soft drinks, packaged breads, breakfast cereals and bars, pre-prepared meals, infant formulas, and meal-replacement shakes [13].

Therefore, this review aims to provide a comprehensive overview of the current knowledge and recent advances related to pesticide residues in food, with a particular focus on strategies for their detection, regulation, and mitigation. The review begins with a historical perspective on the evolution of pesticide use in agriculture, examining the regulatory frameworks governing pesticide residues from both a global and European Union (EU) standpoint. It explores the main analytical methods used for determining pesticide residues in food and presents a detailed classification of commonly used pesticides. Additionally, it identifies the key factors influencing food contamination by pesticide residues, including environmental, chemical, and agricultural variables. Emphasis is placed on both conventional and household-level practices for reducing residues during food preparation and processing, as well as emerging and innovative technologies for pesticide removal. Finally, the review discusses the growing role of Artificial Intelligence (AI) as an indirect but powerful tool for predicting contamination levels and optimizing mitigation strategies, highlighting its potential to transform food safety and pesticide management systems.

## 2. Historical Evolution of Pesticides and Regulations at EU and Global Level

Ever since ancient times, civilization has found ways to cultivate and preserve its food resources, especially in times when they were scarce. Elemental sulfur was once used due to the understanding that a toxic environment could repel insects, and traditional Chinese medicine also uses primitive sulfides. The Ebers papyrus (1500 B.C.E) contains preparation techniques for the removal of insects as well as Homer’s “Odysseus” (800 B.C.E). Around the 1500s, the early stages of what was called “para-pesticides” emerged, which included mercury and arsenic, used until the 1940s [14].

In the 19th century, sulfur compounds were utilized as fungicides, while arsenical compounds were employed as insecticides. A major turning point occurred in the 20th century, particularly in the immediate aftermath of World War II. Due to the high toxicity of sulfur and arsenic-derived pesticides, chlorinated organic pesticides such as dichlorodiphenyltrichloroethane (DDT) and benzenehexachloride (BHC) were developed in the 1930s and widely used in the 1950s and 1960s. These compounds had a long environmental persistence, and thus, farmers hoped to use them to control insects that were previously considered uncontrollable. They were also used in the treatment of malaria and other insect-borne diseases [15].

There were, however, concerns arising due to the toxic effects of DDT on wildlife and humans, as well as its environmental persistence and accumulation in the food supply. A pivotal moment in pesticide history occurred in 1962, when American biologist Rachel Carson published Silent Spring, in which she exposed the dangers of DDT bioaccumulation and biomagnification up the food chain, raising concerns about the long-lasting effects of pesticides on wildlife, particularly human life [16]. In the case of DDT, these pathways led to the near extinction of multiple bird species. Birds that ate contaminated fish absorbed DDT, resulting in the development of thin-shelled and malformed eggs. The introduction of this insecticide into aquatic ecosystems triggered a chain reaction that increased contamination throughout the entire food chain [17].

Silent Spring was responsible for sparking public concern about the improper use of pesticides and the need for better control over their use, launching the modern environmental movement. In 1972, the United States Environmental Protection Agency (EPA) issued a cancellation order for DDT due to the adverse environmental effects and potential human health risks. Ever since then, more research has been conducted to correlate DDT exposure to reproductive effects in humans, based on animal studies. Even though the DDT concentration in the environment has decreased since its discontinuation, its persistence still raises concern. DDT is still recommended by the World Health Organization (WHO) for indoor residual spraying, particularly in African countries where malaria remains a significant public health issue [18].

There is growing concern regarding regulations implemented in food safety. In the European Union, the approval process for pesticides is managed through a unified system, mainly under Regulation [19] (EC) No 1107/2009, which mandates thorough evaluations of human health, safety to the environment, disruptions to hormones, and limits on residues (MRLs) set by [20] Regulation (EC) No 396/2005 and its amendments. The EU adopts a hazard-focused method for approving active ingredients, resulting in the prohibition of many substances that are still allowed in other places.

Different areas implement various systems. In the United States, The Environmental Protection Agency (EPA) oversees regulations under the Federal Insecticide, Fungicide, and Rodenticide Act (FIFRA), using a risk-based approach that considers hazards, levels of exposure, and agricultural requirements. Nations like Canada, Australia, and Japan follow similar risk-based practices, with regular assessments of active ingredients. On a global scale, the FAO/WHO Joint Meeting on Pesticide Residues (JMPR) offers scientific advice on toxicity and MRLs, aiding in the standardization across countries.

The European Union has set bold goals to increase organic farming as part of the European Green Deal and specifically the Farm to Fork Strategy. This policy aims to utilize at least 25% of the EU’s agricultural land for organic farming by the year 2030, backed by the Organic Action Plan that was approved in 2021 [21] (Action plan for organic production in the EU. European Commission, 2021). Recent statistics show steady but not enough progress towards this aim: the amount of land used for organic farming grew from 5.9% in 2012 to about 10.5% in 2022, which is roughly 16.9 million hectares in total across the EU. Even with this overall increase, there are still considerable differences among Member States—countries like Austria, Estonia, and Sweden have already surpassed 20% of their agricultural land for organic use, while some others are still under 5%. These numbers highlight the encouraging upward trend of organic farming in the EU and the significant push needed to meet the 2030 goal.

The EU’s Organic Action Plan is a detailed approach aimed at increasing and enhancing organic farming throughout the Union. It focuses on three primary goals: increasing consumer interest and confidence, assisting farmers in switching to organic methods, and improving the positive impact of organic practices on the environment. This plan aligns with the EU’s Farm to Fork Strategy, which aims for at least 25% of the EU’s farmland to be converted to organic farming by 2030. The plan includes 23 initiatives that promote the use of the EU organic label, increase organic options in public purchasing, enhance traceability, and fight against fraud. To assist with this shift, the plan uses various supportive policy tools like the Common Agricultural Policy, advisory help, and specific funding. It also boosts local supply chains and research funding, ensuring that a minimum of 30% of agricultural research and innovation resources are allocated to organic topics. By focusing on sustainability, it relies on the well-known environmental advantages of organic farming, such as improved biodiversity and decreased use of synthetic materials, backed by revised EU laws that have been effective since 2022 [22].

The European Commission is also implementing the Nature Restoration Regulation, a plan to restore ecosystems by 2050 and halve pesticide use by 2030 [23]. The focus remains on minimizing pesticide residues in food and reducing the ecological footprint of agricultural practices.

To ensure food safety, risk assessments are conducted by the Joint FAO/WHO Meeting on Pesticide Residues (JMPR), which establishes limits for safe intake based on international data and the determined level of risk. These limits are imperative to ensure that the level of exposure through food does not result in adverse health effects over a person’s lifetime. The values determined are then used by governments and international bodies, like the Codex Alimentarius Commission, to establish international Maximum Residue Limits (MRLs) for pesticides in food. The standards imposed by the Codex serve as the reference for international food trade, allowing consumers to be confident that the food they purchase meets the standards for safety and quality [24].

Within the European Union (EU), the regulatory framework is defined by Regulation (EC) No 396/2005, which sets MRLs for all food and feed products (plant and animal origin alike), including imported products [20]. The European Food Safety Authority (EFSA) is responsible for providing risk assessment reviews, in coordination with EU Member States, and advises safe exposure levels [25]. EU regulations follow a precautionary approach, adopting a default MRL of 0.01 mg/kg for pesticides that are not mentioned in the legislation of substances that are non-renewable, where the MRL can be set to the lowest quantifiable level, sometimes lower than 0.01 mg/kg [9].

Over the years, there has been a growing awareness of the ways pesticides can affect humans, directly and indirectly, through multiple routes. Oral exposure to pesticides through food is a significant factor contributing to their toxicity. Although acute pesticide poisoning has become a rare event, chronic toxicity is the one that raises bigger concerns, being responsible for potentiating chronic diseases such as cancer, asthma, endocrine disorders, immunotoxicity, and many others. Disturbances in cellular homeostasis could cause these chronic disorders, the primary action of pesticides, and the accumulation of DNA damage [26]. Due to these concerns, the regulatory authorities, in combination with the European Commission, are in a constant effort to update the EU Pesticides database [27], according to the latest findings, and, in this way, establish the correct MRLs for the different pesticides. These tools allow users to search for information about active substances, approved and non-approved in the EU; food products, and MRLs from pesticides that apply to them; pesticide residues and subsequent MRLs; as well as emergency authorizations in Member States of the EU [27]. It serves as a valuable reference for regulators, researchers, producers, and consumers alike, ensuring compliance and transparency throughout the food chain.

## 3. Methods to Determine Pesticide Residues in Food

Accurate detection and quantification of pesticide residues in food are essential for ensuring compliance with safety standards and protecting public health. Over the years, analytical techniques have evolved to become more sensitive, efficient, and capable of detecting multiple pesticides simultaneously.

One of the most used sample preparation methods is QuEChERS (Quick, Easy, Cheap, Effective, Rugged, and Safe). It involves a straightforward extraction with acetonitrile, followed by a cleanup step using dispersive solid-phase extraction. Initially, the method was developed for food matrices with high water and low-fat content, but after modification, it could be applied to dry and fatty foods. Its advantages include simplicity, good accuracy, reducing analysis time, and compatibility with a broad range of pesticide types [28]. Some changes in the original QueChERS method has also been used with effective results for multiple pesticides [28].

For the detection and quantification phase, Liquid Chromatography coupled with Tandem Mass Spectrometry (LC-MS/MS) has become the gold standard. However, GC-MS/MS is also frequently used, especially for organochlorines, organophosphates, and pyrethroids. These methodologies provide highly sensitive and specific testing, even in complex food samples, enabling the determination of pesticides from different chemical families in a single run [29]. The tandem mass spectrometry component provides structural information based on the MS/MS spectra present in the system database, which contributes to the correct identification of the compound, even in cases where raw mass spectra overlap with those of other substances [30].

The European Union’s SANTE guidelines provide guidance on the validation, quality control, and analytical performance criteria for pesticide residue analysis in food and feed. The guidelines recommend the use of methods like QuEChERS for sample preparation and by GC or HPLC/UPLC coupled with mass spectrometry (MS) for identification and quantification. Various MS systems (e.g., quadrupole, ion trap, TOF, orbitrap) and ionization techniques (EI, CI, APCI, ESI) may be employed, using acquisition modes such as full-scan, SIM, SRM, or MRM [31]. The criteria defined by this guideline, which strongly support the use of these two techniques, allow researchers to have confidence in the validation of the methods used [32].

## 4. Processing Factors

Many foods undergo processing before they are consumed, which can significantly influence the levels of pesticide residues. To account for changes in pesticide residue levels resulting from processing, the concept of processing factors (PFs) is employed. A processing factor is defined as the ratio between the residue level in a processed product and that in the corresponding native agricultural product. When PF is greater than 1, it means that residues are concentrated in the processed product, such as during drying or oil extraction. If PF is less than 1, there is a decline in the residues in the processed product, such as through washing or boiling processes [33].

Processing characteristics are critical in assessing food exposures. Among these, generic processing parameters are linked to specific processes, such as dehydration or dilution, and help explain how pesticide residues behave throughout food processing. These differences must be recognized, especially for meals taken in processed forms. For example, drying grapes into raisins increases residue concentrations due to water loss, whereas peeling an orange lowers residue levels, as the majority of residues are contained in the peel [33].

Within the EU, the European Food Safety Authority (EFSA) plays a central role in evaluating pesticide residues and processing factors, releasing the first version of the “EU database of processing factors for pesticide residues” in 2018. The current update of the database contains 2838 processing studies, which allows EFSA to present 19,384 individual and 4564 median processing factors. These processing studies allow EFSA to support risk assessments and refine consumer exposure estimates [34]. Although Regulation (EC) No 396/2005 does not fix legal MRLs for processed products, it does require, under Article 20, for the Authority on MRLs to maintain and update a database with processing factors, containing dietary intake assessments [20], seeing as processing factors could be used through extrapolation, to support safety evaluations if necessary [33]. These factors are also included in models like EFSA PRIMo (Pesticide Residue Intake Model), which estimates short- and long-term dietary exposure to pesticide residues, used as a prospective screening tool for dietary risk assessment [35].

This way, processing factors are crucial tools, helping regulators and scientists to determine how food processing affects pesticide levels and contributes to ensuring that consumers are not exposed to harmful amounts.

## 5. Classification of Pesticides

**Figure 2:**
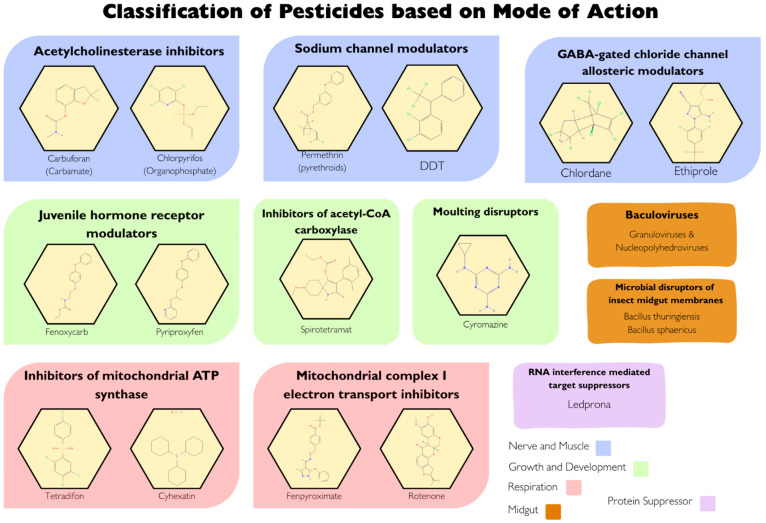
IRAC Pesticides Classification based on the Mode of Action.

Pesticides can be classified according to different criteria [36,37,38]. Based on how toxicity they are, they are divided into categories from very poisonous to those with low toxicity for people and animals. Based on their function, they are classified according to the specific pests they deal with, like insecticides, fungicides, herbicides, or rodenticides [39]. According to their chemical composition, pesticides are organized by their chemical structure, which includes categories such as organophosphates, carbamates, pyrethroids, and triazoles [40,41]. Depending on how they get to the target organism, they are categorized by their method of entry, including contact, systemic, stomach poisons, or fumigants [42]. Finally, according to mode of action they are grouped by the biological functions they interfere with, such as affecting the nervous system, blocking breathing, hindering growth, or disrupting cell membranes or walls (Figure 2) [43]. classification of pesticides helps clarify why different compounds behave differently during application, persistence, and removal. By grouping pesticides according to their chemical class, mode of action, or physicochemical properties (such as solubility, volatility, and stability), the classification highlights the key factors that influence how strongly residues bind to crops, how long they remain, and how easily they can be degraded or removed. For example, highly lipophilic pesticides tend to adhere more strongly to waxy surfaces, making them harder to wash off, while more water-soluble pesticides may be more responsive to rinsing or aqueous decontamination methods [41,42]. Understanding these distinctions allows for better prediction of residue behavior and for selecting the most effective and targeted residue-removal strategies.

## 6. Factors That Most Influence Contamination of Food with Pesticide Residues

### 6.1. Physicochemical Properties of the Pesticide and Its Formulation

The inherent characteristics of an active substance—water solubility, Log Kow, vapor pressure, and photolytic/biodegradation half-life—largely determine how long residues persist. Pesticides with high vapor pressure are preferred as fumigants, such as thymol, due to their useful penetrative power; however, there is a danger of environmental pollution due to vapors [44]. Highly lipophilic compounds, such as organochlorine pesticides, can accumulate in fatty tissues or waxy cuticles, exhibiting high persistence in food and the environment [45]. Formulation additives (wetting agents, micro-encapsulation) considerably influence pesticide persistence by prolonging surface residence and slowing degradation [46].

### 6.2. Application Practices and Compliance with Good Agricultural Practice (GAP)

Residue levels are strongly influenced by what, how, and when a pesticide is applied. In this context, several parameters must be considered:

(i) Product choice and dose. Using an active substance not registered for a specific crop or applying it at a rate exceeding the label recommendation greatly increases the likelihood of exceeding the maximum residue limits (MRLs) [47]. Conversely, using adjusted doses is consistent with the principles of good agricultural practices (GAP) and with Regulation (EC) No. 396/2005, as it promotes lower residue levels in food and compliance with distribution requirements [48], (ii) Frequency and pre-harvest interval (PHI). Skipping or shortening the PHI leaves insufficient time for residue dissipation [49]. The PHI can also vary depending on the growth stage at the time of application, which affects pesticide deposition and dissipation dynamics. These factors are directly related to achieving compliance with MRLs [50], (iii) Application technique. Poorly calibrated sprayers, aerial spraying under windy conditions, or high-pressure ground equipment can produce fine droplets that shift from the intended target, landing on adjacent crops or plants. The type of chemical applied, its mode of action, and the label requirements must also be considered [51]. Drift losses to non-target areas have been estimated to be between 50 and 60%, resulting in economic losses and undesirable crop contamination [52].

### 6.3. Crop Morphology

Morphological traits, which relate to the physical structure and appearance of plants, such as size, shape, and architecture [53], will affect the distribution of pesticide residues. The height and shape of plants has a significant impact on the dispersion of residues, with vegetables grown near the ground, such as leafy greens, being handled more consistently than higher crops [54]. The position of the fruit in the tree is also a factor to consider during pesticide spraying, as fruits at the middle and bottom of the tree are more susceptible to having higher residues than those on the top parts of the tree [54]. Berries, like strawberries, blackberries, blueberries, and cherries, and leafy greens, like spinach and kale, are some of the produce that appear in the 2025 “Dirty Dozen”, a list of the most contaminated fruits and vegetables [55]. Compared to other vegetables, leafy vegetables have a large leaf surface area, combined with a thin waxy epidermis and an increased number of stomata, which leads to more favorable absorption and translocation of pesticide residues [56].

### 6.4. Environmental and Edaphic Conditions

Various environmental factors influence pesticide degradation and, consequently, the levels of residues that reach crops. Among these factors, temperature, soil organic matter, solar radiation, pH, and rainfall play a major role in determining the rate and pathway of pesticide dissipation [57,58]. Temperature affects the molecular stability of pesticides, altering their degradation rates in the environment. Typically, degradation increases under higher temperatures and slows down under cooler conditions [59]. Soil organic matter can help increase pesticide degradation due to enhanced microbial activity; however, it can also decrease it by increasing the adsorption of pesticides onto the soil, which can cause their transfer to other crops and water bodies [59]. Solar radiation is a crucial element for adequate crop growth and also influences photodegradation processes. The degree of this effect relies on the intensity of light and the duration of exposure [60]. Finally, heavy rainfall shortly after spraying can wash residues into the soil, especially for pesticides with high water solubility, which can result in groundwater contamination [60].

### 6.5. Post-Harvest Treatments and Storage

Residues are not always field-derived. Some farmers and market vendors use pesticides during the post-harvest period as a way to extend the shelf life of fruits and vegetables [61].

One of the most common examples of pesticide use in the post-harvest phase is the use of sprout inhibitors in potatoes, which helps prevent food waste and loss of revenue [62]. The most used sprout suppressant is chlorpropham (CIPC). This carbamate is inexpensive and highly efficient, with a single application being sufficient to inhibit sprouting for up to 5 months due to long-lasting residues [62]. This, however, can also be seen as a concern since the residue will not dissipate completely before reaching the consumer. Mammal digestive activity of CIPC produces 3-chloroaniline (3-CA), a toxic compound that targets the hematopoietic and renal systems, and that can be potentially dangerous to humans [62]. In 2025, almost 90% of U.S. potato samples contain chlorpropham, despite the EU banning it in 2019 [63].

In post-harvest insect pest management, while alternative methods such as cold treatments, heat treatments, irradiation, controlled-atmosphere storage, and fogging are utilized, the application of fumigants remains a crucial option. For quarantine and pre-shipment disinfestation of horticultural products, methyl bromide has historically been the main selection. Nonetheless, because of its potential to deplete ozone, its application has been gradually limited, and phosphine is now the permitted fumigant for citrus treatment in the United States [64].

At any stage of the food chain, post-harvest handling and the selected processing methods can significantly affect the concentration of pesticide residues [61].

### 6.6. Household Preparation and Processing

Household activities such as washing, peeling, blanching, juicing, cooking, milling, and oil extraction can lead to changes in pesticide levels [61]. Peeling is very effective at removing surface residues of non-systemic pesticides that do not penetrate plant tissues, but it is less effective against systemic compounds. Boiling reduces pesticide residues mainly through evaporation, hydrolysis, and distillation processes [65]. Systemic pesticides, however, are more challenging to eliminate because they penetrate deeper into plant tissues, making them less susceptible to removal during processing [66]. Organochlorine pesticides are recognized as lipophilic substances, dissolving in organic solvents but not in water, which makes them more likely to accumulate in fatty tissues [67].

### 6.7. Regulatory Oversight and Socio-Economic Drivers

The food and agriculture industry faces challenges when MRLs become more restrictive before food clears the trade channels. Stored food and agricultural products, like nuts, dried fruits, and juices, can become non-compliant due to changes in the MRLs after storage [68]. In the Chinese national surveillance dataset, a 1% increase in official sampling frequency was associated with a 0.28% decrease in over-MRL detections, underscoring the deterrent effect of enforcement [69].

There is also the issue related to the cosmetic standards of food that are applied by buyers, causing producers to make wrongful use of pesticides as a way to achieve these standards and corresponding price incentives, and prevent produce loss [70].

### 6.8. Global Supply Chain and Trade Patterns

Producers who wish to export their produce face a reality of different MRLs for the same crop in different markets, including when an MRL is low or missing [71]. There is also the issue of different authorized pesticides, which leads producers to find alternative pesticides or other alternatives to sell their produce in the market, resulting in additional expenses and the introduction of unwanted residues into the food chain [71]. The length of the logistic chain leads to the use of post-harvest fungicides as a way to prevent food losses, especially in fresh produce [72]. Sprout-inhibitors are used to prolong shelf-life and maintain the desirable appearance and nutritional quality of the produce [73]. Efforts have been made to harmonize pesticide regulations, with the Codex Alimentarius Commission (CAC) establishing maximum residue limits (MRLs) for various types of produce, referred to as Codex MRLs. Nevertheless, the level of conformity with these MRLs varies globally [74]. This variation can occur because certain countries overlook foreign Good Agricultural Practices (GAP) when they already have domestic standards, or because Codex MRLs are enforced solely on products meant for export [75].

The contamination by pesticide residues is a multifaceted phenomenon: chemical structure, agronomy, environment, and market forces are all responsible for the propagation of residues in the food chain and ecosystems [60]. This way, there is a necessity for effective mitigation strategies, which require an integrated technology—integrated pest management (IPM), precision application technologies, farmer training, and vigilant post-harvest control [76,77,78]—in tandem with constant monitoring and the harmonization of standards, to ensure that food reaches the final consumer within the proper safety limits.

In the next section, special emphasis will be given to methods for reducing pesticide residues in food during household preparation and processing.

## 7. Strategies for the Mitigation of Pesticide Residues in Food Through Household and Processing Practices

Given the widespread use of pesticides in agriculture, there is increasing concern about the residues that end up in our homes, particularly on foods consumed raw. Consequently, one of the key questions is: what is the most effective method for removing pesticide residues during household food preparation? In this section, various processing methods, including washing and peeling, will be addressed.

### 7.1. Washing

The most common and simple method of household processing is washing our produce before cooking or eating it, when raw. However, studies show that even the method of washing, using simple running water, with the aid of a device, or by adding detergent or disinfectant, makes a difference in the final concentration of pesticide residue. Washing efficiency depends on the pesticide concentration, washing time, and the extent of rubbing of the produce [79] (Table 2).

A study conducted in South Korea, which measured the reduction in pesticide residue in five leafy vegetables (lettuce, perilla leaves, spinach, crown daisy, and ssamchoo) using nine different washing methods, showed varying percentages of reduction for all samples and methods alike. The study analyzed the rate of reduction of ten different pesticides, such as azoxystrobin, chlorantraniliprole, chlorfenapyr, diniconazole, fludioxonil, imidacloprid, indoxacarb, lufenuron, pyraclostrobin, and thiamethoxam. The vegetables were processed with running tap water, stagnant tap water, ultrasonic cleaning, vinegar, sodium bicarbonate, vegetable detergent, blanching, and boiling. After analyzing the results, they concluded that the most efficient method was washing with running tap water (77%), while the least efficient method was using vegetable detergent (43.7%). Azoxystrobin was the pesticide residue with the highest reduction (66.2%), while lufenuron was the pesticide with the lowest (45.1%) [80].

Another study, conducted in Portugal, measured the efficacy of washing methods in brown, long-grain, and basmati rice using water alone and a mixture of water with apple cider vinegar. The samples were spiked with concentrations of 20 μg/kg and 50 μg/kg of 121 different pesticides. For the long-grain rice, washing with water resulted in a reduction of up to 30.4% and 73% (20 μg/kg and 50 μg/kg, respectively), while washing with apple cider vinegar presented results of up to 39% and 80.3% reduction. For brown rice, washing with water resulted in a reduction of up to 7.5% and 53.4%, while washing with vinegar presented results of up to 58.8% (50 μg/kg), with some of the pesticides below their limit of quantification (LOQ) in the contamination level of 20 μg/kg. Finally, for the basmati rice, washing with water showed a reduction of up to 51.3% for the higher concentration and a nearly insignificant value for the lower concentration, while washing with vinegar reduced the residues by 63.2% in the higher concentration and below the LOQ in the lower concentration. The study concluded that washing with the vinegar solution yields better results and highlighted the difference in elimination rates due to the varying composition of the rice [81].

A study made in China measured the efficiency of washing tomatoes under tap water for 5 min. It achieved a removal rate of up to 80% during the washing process, taking into consideration that the pesticide solubility in water has an impact on the degradation. The pesticides used in this research were paclobutrazol, tebuconazole, prothioconazole, cyflumetofen, difenoconazole, triadimefon, triadimenol, and difenoconazole-alcohol (CGA 205375). The pesticides in this experiment were below their maximum solubility in water [82].

### 7.2. Peeling, Milling, and Polishing

Peeling is a common method of in-home food preparation, involving the removal of the peel from fruits and vegetables, often before consuming them raw or preparing them for further processing. Peeling is a simple method for removing pesticides. However, it cannot completely eliminate them; it can also cause nutritional loss due to the antioxidants and bioactive compounds found in the peels of fruits and vegetables [79]. Studies were conducted to determine whether removing the peels of fruits and vegetables had an impact on reducing pesticide residues.

A study conducted in Poland assessed the permeation of pesticides through an apple peel after spraying for up to 24 h to understand if peeling the fruit would have a significant impact on reducing the amount of residue present [83]. The research study demonstrated that the peel functioned as a membrane, composed of distinct layers that were permeable to various components based on their characteristics. The outermost layer was permeable to lipophilic compounds but retained polar and water-soluble compounds. However, it also showed that pesticides were able to permeate the pulp through small cracks caused by the maturation of the fruit. The results showed that cypermethrin (a lipophilic pesticide) presented the lowest amount of penetration, with the mean concentration values below the LOQ, while pirimicarb (a hydrophilic pesticide) presented the biggest amount of penetration, with a mean concentration value of 23 mg/kg. Overall, the study concluded that some pesticides could permeate through the peel and into the pulp, meaning that common techniques such as washing and peeling may not be sufficient to reduce the residues of pesticides present [83].

A separate study conducted in China investigated the distribution of pesticides between the peel and pulp of grapes. Twenty-five pesticides were evaluated and categorized into four groups according to their level of migration.

Groups A (pyrethroids, pp’-DDE, and chlorfenapyr) and B (difenoconazole, azoxystrobin, and iprodione) comprised pesticides whose residues primarily stay on the peel or have limited transfer to the pulp (up to 20%) [84]. Pyrethroids are recognized for their low toxicity in humans, but poisoning may happen via the disruption of sodium and chloride channels. pp’-DDE possesses a high fat-water partition coefficient and tends to accumulate in fatty tissues. Chlorfenapyr disrupts ATP production by uncoupling oxidative phosphorylation in mitochondria, resulting in cellular dysfunction. Difenoconazole is a broad-spectrum fungicide that inhibits the demethylation process in ergosterol production. Azoxystrobin inhibits mitochondrial respiration in fungi, while iprodione acts as a contact fungicide, blocking mycelial development and spore germination [41,85,86,87,88,89]. Grapes treated with these pesticides can typically be consumed safely after proper washing and peeling (although peeling by mouth is not advised) and are deemed safe for winemaking, as storage facilitates some breakdown of residues. Groups C (dimethomorph and cyprodinil) and D (pyrimethanil and metalaxyl) consisted of pesticides that can significantly migrate into the pulp, rendering the grapes unfit for consumption despite washing and peeling [84]. Dimethomorph and cyprodinil are fungicides that work throughout the plant. Dimethomorph belongs to the morpholine group and prevents the creation of ergosterol. Cyprodinil stops the production of methionine and interferes with the growth of mycelium. Pyrimethanil is applied to control grey mold on fruits and vegetables, while metalaxyl is a systemic fungicide that effectively combats water mold fungi [84].

An investigation was conducted to determine if consuming fruits (apples, pears, and peaches) with the peel had any significant nutritional value and what risks were associated with it, especially related to the pesticide residues present (mancozeb, malathion, and endosulfan, respectively) [85]. The analysis concluded that peeling could be an easy and efficient method for removing residual pesticides, especially when combined with a post-peeling wash, by eliminating any residues that could have passed into the pulp. However, this second wash will cause the partial loss of nutrients, vitamins, fiber, and minerals present in the fruit, which are important for human health. For apples, peeling resulted in a 48% decrease in ascorbic acid; pear peel is high in arbutin, a compound with anti-inflammatory and antibacterial activity, used in urinary therapy; and peaches, whose peel contains malic acid, carotene, dietary fiber, phenolic acids, and flavonoids, that decrease by more than 20% if the peel is discarded [85].

A significant part of the global population’s energy source comes from cereal grains and rice. Due to this, there is a necessity to process them before human consumption, to transform them into palatable, nutritious, and convenient food products [90]. One of the processing steps that grains undergo is the polishing process, which helps remove dust, spores, fungi, and other contaminants that adhere to the grain [86].

There is also the milling process, which is used, for example, in rice processing, yielding different rice products through different degrees of milling (DOMs), from brown rice (0 DMO) to polished rice (12 DOM). Research was conducted to determine if the milling process, using different degrees of milling (DOM), had any effect on the reduction in pesticide residues in etofenprox, flubendiamide, and tebufenozide. The results showed that milling rice from brown to polished rice cause a 93.16% reduction in etofenprox, 90.25% reduction in flubendiamide, and a 92.58% reduction in tebufenozide, thus concluding that the more rice is milled, the greater is the reduction in pesticide residues, especially taking into consideration that the pesticides used in this study were non-systemic pesticides and therefore remained in the surface of the grain [87].

### 7.3. Drying and Dehydration

Drying is a frequently used method to reduce post-harvest loss and extend the shelf life of fruits and vegetables by removing moisture, thereby preventing the growth and reproduction of microorganisms. This technique can lead to a reduction in pesticide concentration due to evaporation and thermal degradation, especially in the case of water-soluble pesticides, which are more susceptible to this type of change. However, there are also cases in which drying can cause an increase in pesticide residues, due to the consequent dehydration that leads to water loss and weight changes. Additionally, degradation could lead to the formation of toxic metabolites, such as carbofuran, a neurotoxic metabolite of carbosulfan derived from pesticide oxidation, hydrolysis, and photolysis [88].

A way to avoid the possible increase caused by drying and dehydration is to combine this technique with other processes, such as a previous wash, to achieve a prior reduction of residue [89].

### 7.4. Juicing and Oil Extraction

Fruits are often processed into juice as an alternative means of consumption, particularly when they are overly ripe or less appealing to eat fresh. The juicing process typically involves several stages, including washing, peeling, and filtering the pulp. Numerous studies have evaluated whether juicing effectively reduces pesticide residues in fruits. It is essential to recognize that the presence of pesticide residues in fruit juices is influenced by the distribution of residues in the peel and pulp, as well as their physicochemical properties. Additionally, processing steps such as centrifugation and filtration can also help reduce the levels of residue. Pesticides with low water solubility are easier to remove than pesticides with a higher solubility, which end up being transferred into the juice [91].

During juice production, clarification is essential for removing solids from the liquid phase, which also helps eliminate pesticide residues present in the solid portion of the product. A study investigated the effects of clarifying agents on the removal of pyrethroid residues in apple juice [92]. Flour clarifying agents (bentonite, casein, gelatin, and polyvinylpolypyrrolidone (PVPP)), in different doses, were added to samples of apple juice purposely enhanced with pyrethroids. Bentonite, casein, gelatin, and polyvinylpolypyrrolidone (PVPP) were added at different concentrations (50, 150, 250, 500, and 750 mg/L) to apple juice spiked with pyrethroids.

The results showed that removal efficiency varied with both the type of agent and its dose. Medium doses generally provided optimal removal, whereas low (50 mg/L) and high (750 mg/L) doses were less effective due to saturation of the adsorbent surface and excessive accumulation, respectively. Overall, gelatin was the most efficient agent, with an optimal dose of 500 mg/L, while casein had the lowest efficiency. Although both are proteins, their differing amino acid compositions explain this discrepancy; gelatin contains more hydroxyl and amine groups, enhancing interactions with pyrethroids in the juice [92].

Aside from juicing, it is also possible to extract oil from certain fruits and vegetables. Oils are often used for cooking and processing, such as blended oil, a mixture of vegetable oils (peanut oil, sunflower seed oil, soybean oil, etc.), commonly used in China [93]. One of the most commonly extracted oils is olive oil, which is the main source of lipids in the Mediterranean diet. The quality of the olive oil depends on the quality of the olive crops, and these trees are prone to diseases caused by insects and weeds. Therefore, pesticides are applied to control pests and improve the quality. This raises concerns about residues that may accumulate on the oil, although monitoring is challenging due to the high triglyceride content [90]. As the most desirable oil is extra-virgin olive oil, which is produced only through mechanical extraction and without any other refining processes, there is a possibility that it may retain pesticide residues [94]. To ensure that the oils are safe for consumers, it is important to follow the maximum residue level (MRL) established in Regulation (EC) No 396/2005, which attributes an MRL to the olive, not its final product (oil) [22]. For this, a processing factor adapted to the class of the pesticide in question, in which fat-soluble pesticides multiply the MRLs by a factor of 5 [94].

### 7.5. Jam-Making

Another alternative for the full use of a fruit is to turn it into jam. The heating treatment, combined with the addition of sugar, creates a mixture that, if bottled using the correct techniques (vacuum sealing), can be preserved for a long time. A few studies have been conducted to investigate whether jamming is a viable method for reducing pesticide residues at home.

In the process of making homemade jams and marmalades, fruits are often boiled beforehand to soften the pulp. A study, using homemade orange jam, boiled the fruit three times before adding the sugar, and changing to fresh boiling water each time [91]. Five pesticides were used for this study: abamectin, buprofezin, ethoxazole, imazalil, and thiophanate-methyl. Abamectin, ethoxazole, and thiophanate-methyl were not detected in the jam, as their concentrations were below the limit of determination (LOQ) of 0.01 mg/kg. Buprofezin and imizalil decreased by 90% and 95% of their initial levels. They concluded that this process could be responsible for removing water-soluble pesticides through boiling, evaporation, heat degradation, and the temperature of the heat treatment, as well as processing time. It was also noted that the chosen cooking technique and the physicochemical structure of the residue, especially its solubility in water and volatility, could impact the residue present in the final product [91].

### 7.6. Blanching

Blanching is a mild heat treatment applied to food before further processing methods such as drying, freezing, frying, or canning. While it is not considered a preservation technique in its own right, blanching can reduce the microbial load on fruits and vegetables to some extent. The process involves briefly exposing the produce to hot water or steam at temperatures between 90 °C and 95 °C for about 1 to 2 min. Immediately afterward, the food is rapidly cooled—typically by immersion in ice water or by spraying with cold water—to stop the cooking process. Blanching is primarily used to soften the texture of produce through partial cooking and to reduce strong or bitter flavors [95] (Table 3).

Blanching spinach for 2 min resulted in a decrease of up to 41% (propamocarb) of the pesticide residues, a very positive value compared to washed samples, which only showed a reduction of 8%. When increasing the processing time to 6 min, the results showed a decrease of up to 44%, but a concerning increase of up to 28% (fluopicolide), in contrast with washing (−47%) [96]. The study concluded that the optimal blanching time was 2 min at 80 °C, as it showed the best positive results. The short time also helps reduce the effects of the process on the vegetable structure [96].

Blanching cowpeas in boiling water for 2 min resulted in a significant removal of up to 67.6% (cyromazine), with the 5 min process having a similar effect on removal. For the remaining three pesticides included in the research—acetamiprid, chlorantraniliprole, and thiamethoxam—the results showed similar efficiency, except for thiamethoxam, which achieved a 49.8% removal rate. In contrast, acetamiprid and chlorantraniliprole achieved removal rates of 60.9% and 66.4%, respectively. When combining washing for 40 s with blanching for 2 min, the process was able to reduce the residues to below the maximum residue level (MRL) concentration. This way, the study concluded that the 40 s wash followed by a 2 min blanch was a safe processing method for consuming cowpeas [97].

Regarding the effects of 2 min blanching on removing residues of carbamates, organophosphates, and pyrethroids from Chinese kale and yard-long beans, a study yielded different results [98]. There was a moderate removal of the carbamates, up to 69%, while organophosphates were only 20% removed, and pyrethroids also had a moderate removal of up to 45%. In the case of yard-long beans, the results were similar, with organophosphates having a low removal (27%) and pyrethroids with a moderate removal of 59%. The investigators concluded that cooking practices, such as blanching, can help reduce pesticide residues in vegetables when combined with washing, thereby encouraging the consumption of vegetables [98].

### 7.7. Thermal Processing Techniques

Thermal processing techniques involve subjecting fruits and vegetables to the effects of different temperatures. Techniques such as boiling, cooking, and frying are included in this method [64].

For instance, frying apples with the skin at 150 °C caused a 92.9% reduction in deltamethrin and an 88.5% reduction in fludioxonil, two non-systemic pesticides [66].

Boiling Chinese kale for 10 min resulted in a 71% removal of indoxacarb, a carbamate, but showed lower efficiency for the other two carbamates, carbofuran (35%) and fenobucarb (21%). It had a moderate effect on pyrethroid pesticides, such as deltamethrin (66%), fenvalerate (62%), and cypermethrin (56%). Still, it had a minimal effect on the removal of flumethrin (9%), which could be justified by the compound’s higher lipophilic structure [99]. Boiling can also help eliminate volatile pesticides due to the heat, and hydrolysis-prone pesticides are reduced in this method as well [100].

### 7.8. Fermentation

A newly explored method for mitigating pesticide residues is the use of microbial fermentation. Foods’ own microflora or added probiotic strains can be used to metabolize synthetic insecticides through enzymatic degradation, utilizing them as a carbon and energy source. Two of the main microorganisms used for this type of processing are lactic acid bacteria (LAB) and yeast strains. Studies have shown that the use of the fermentation process in vegetables, which are usually prone to residual organophosphate pesticides, can help decrease residues. Specifically, chlorpyrifos is completely degraded after nine days of kimchi fermentation [101].

This technique is also employed in the production of alcoholic beverages, such as wine, where Saccharomyces cerevisiae is used as the yeast for the fermentation process. This way, we could question if these residues could have any impact on the fermentation process itself. Research was conducted to understand the impact of residues from five pesticides (tebuconazole, difenoconazole, azoxystrobin, pyraclostrobin, and thiamethoxam) on wine fermentation, specifically the effect these pesticides have on inhibiting yeast proliferation. The results showed that difenoconazole can delay Saccharomyces cerevisiae growth cycle, at the lowest exposure level of 1 mg/L, decreasing the optical density at 600 nm (OD_600_) by 29.13% on the 7th day of sampling, and cause full inhibition at the exposure levels of 10 mg/L and 25 mg/L (OD600 decreased by 94.32%). Tebuconazole, pyraclostrobin, and azoxystrobin also caused some inhibition, while thiamethoxam did not exhibit an inhibitory effect. According to the researchers, these results could be explained by the type of pesticide and its mode of action. Difenoconazole and tebuconazole are triazole fungicides that inhibit cytochrome P450 51, thereby affecting ergosterol synthesis and, consequently, the integrity of the fungal cell. This way, the difenoconazole inhibition of Saccharomyces cerevisiae could be due not only to its mode of action, but also to the compound’s high lipophilicity, which allows it to accumulate in the cells [102].

### 7.9. Innovative Approaches for Pesticide Residue Removal

Beyond the conventional strategies listed previously, more innovative approaches are being researched as ways to remove pesticide residues with greater efficiency. Although these emerging methods offer distinct advantages, each also presents certain limitations, as summarized in Table 4.

Aside from the conventional methods of washing, the hypothesis of using Electrolyzed Water Devices (EWDs) was investigated in lemons, cucumbers, and carrots to evaluate whether such devices could remove pesticide residues (malathion, fenitrothion, and p,p′-DDT) [110]. These devices operate based on water electrolysis, producing reactive hydroxyl radicals, and utilize ultrasound and UV radiation for additional enhancement. The results showed that after treatment with the EWDs, the amounts of malathion and fenitrothion in lemon peel decreased by up to 40%, in carrots by more than 50% of the malathion content, and in cucumbers by more than 60% of the fenitrothion content. Comparing the use of EWDs with the traditional methods showed a significant decrease in the number of residues left, except in the case of DDT, where the use of water and detergent proved to be the most efficient method [110].

The use of ozone-infused water can be a way to reduce pesticides due to the properties of degradation by hydrolysis, photolysis, and reduction-oxidation [111]. A study investigated the removal of acetamiprid, malathion, and emamectin benzoate with the help of ozonated water and ozonated air in peppers, comparing the detected concentration after removal with the harvest concentration. Ozonated water showed the best results, with reductions of 70.08%, 84.80%, and 100% of the respective pesticides, while ozonated air was unable to eliminate any residue. The treatment with ozone also helps preserve the weight and color of the peppers during storage [112].

Ultrasonic water baths can also be used to reduce pesticide residues. It is a non-thermal technique that shortens processing time, enhances efficiency, consumes less energy, and maintains the nutritional value of fruits and vegetables. There are two types of ultrasonic equipment: sonotrodes and ultrasonic water baths [88]. A research study was made to investigate the removal of chlorothalonil, pyrazophos, and carbendazim from pakchoi through ultrasonic treatment. After treating the leaves under ultrasonic conditions for 10 min, the removal rates of carbendazim, chlorothalonil, and pyrazophos residues were 24.63%, 74.86%, and 45.68%, respectively. The optimal treatment condition was achieved at a frequency of 25 kHz and an ultrasonic power of 0.45 W/cm^2^ at 20 °C. Although the treatment was effective in removing the residues, some physical damage was observed in the analyzed leaves, which was correlated with the intensity of the ultrasonic frequency. This helped determine that the ultrasonic frequency and input power are two critical parameters that need to be considered to prevent damage and subsequent loss of nutritional value [113].

Cold plasma, a glow discharge composed of reactive chemical species at near-ambient temperature, can inactivate microorganisms through the combined effects of heat, electric fields, UV photons, atomic oxygen, metastable oxygen molecules, ozone, and hydroxyl radicals [114]. A research investigation assessed the elimination of malathion and chlorpyrifos residues from lettuce leaves, noting the most significant reduction at 80 kV for 180 s, with declines of 59% for malathion and 57.9% for chlorpyrifos. The treatment showed no significant changes in leaf color; however, extended exposure duration had an adverse effect on decreasing ascorbic acid levels. Consequently, the ideal treatment condition was determined to be 80 kV for 120 s, leading to reductions of 53.1% and 51.4% for malathion and chlorpyrifos, respectively. The research also demonstrated that cold plasma treatment can inhibit microbial growth, thereby prolonging the shelf life of the produce [115].

## 8. Artificial Intelligence (AI) Applied to the Mitigation of Pesticide Residues in Food

Artificial Intelligence (AI) is emerging as a tool that could aid in mitigating pesticide residues in food. AI, associated with technologies like Machine Learning (ML) and Artificial Neural Networks (ANN), is likely to play a significant role in managing environmental pollutants, such as pesticides. AI can help detect different analytes using algorithms capable of assessing and interpreting datasets of spectral, chemical, and physical properties, through quick and precise techniques that are non-invasive and non-destructive [116]. The integration of AI in food safety protocols can help increase detection and response, and provides a prospect for future real-time monitoring [117].

### 8.1. Precision Agriculture and Smart Pesticide Application

Precision agriculture is a management strategy that, utilizing several technologies, focuses on observing, measuring, and responding to variability in crops, fields, and animals, thereby increasing crop yields and animal performance while lowering costs and optimizing process inputs [118]. Within agricultural systems, the Digital Twins (DTs) represent the virtual version of a farmer’s corresponding farm, with real-time data collected by sensors and crewless aerial vehicles (UAVs), which help in the improvement of decision-making and productivity, allowing farmers to monitor, simulate, and optimize farming operations [119]. UAV-based DT models can help improve yield stimulation and disease detection through high-resolution imagery obtained and processed using AI analytics, which aid farmers in informed decision-making. The remote sensing technology can immediately detect infections and damage, monitoring the crop’s health and identifying stress conditions [120]. AI-powered systems can enhance efficiency by utilizing drones, sensors, and machine learning models for automated monitoring, pest identification, and infestation assessment, as well as the subsequent application of control strategies [121].

By being focused only on targeted areas, these AI-driven systems can significantly reduce the overall pesticide load and thereby limit residues on harvested produce.

Nonetheless, the broad use is restricted due to a lack of sufficient data, particularly in labeled pest and disease collections that showcase various growth phases, lighting situations, and farming environments. The ability to transfer models from one area to another remains limited, and a clear understanding of these models is essential for farmer confidence and protection. Additionally, for automated spraying systems to be accepted by regulators, there must be a clear rationale behind the decisions made and strong evidence of effectiveness in real-world settings [122]. 

### 8.2. Predictive Modeling of Pesticide Residue Dynamics

AI can help analyze the data needed to manage disease and pest monitoring, accurately apply fertilizers and pesticides, determine the pre-harvest interval (PHI), and control pesticide residues, all while complying with maximum residue limits (MRLs). This is achieved through the use of drones and intelligent sprays. Neural networks and machine vision technology can be used to detect defects (on the surface or even internally) in fruits and vegetables, allowing for the monitoring of plant pests and diseases [123]. Machine learning algorithms, such as Artificial Neural Networks (ANNs), can be used for pest classification through image recognition, allowing for more selective pesticide use and subsequent residue reduction [124]. The combination of Convolutional Neural Networks (CNN) with other machine learning algorithms, such as support vector machines (SVM), enables a more robust predictive system. This approach allows for cross-checking of the results from the main system (CNN) and presents additional perspectives, thereby strengthening the reliability of the detection system. This integrated approach enabled a comprehensive analysis of the detected pesticides and their respective concentrations [125].

These types of models, such as dynamiCROP, can also be used to analyze the patterns of pesticide dissipation, enabling the control of the final residue through predictions made by the models and assessing the health risk related to pesticide exposure [126].

Main difficulties involve a shortage of good quality experimental data, absence of uniform protocols, and differences brought about by soil types, formulations, and weather patterns. Regulatory agencies remain hesitant to substitute conventional degradation tests with black-box machine learning models, which highlights the need for explainable artificial intelligence for future approval. Combining mechanistic and machine learning methods appears to be a valuable way to address this issue by incorporating physical laws into machine learning systems [119]. 

### 8.3. AI-Assisted Food Sorting and Screening

Advanced AI techniques are being applied in food processing to distinguish between safe and unsafe produce. Biosensors are being explored for use in detecting foodborne pathogens, thanks to the combination of a biological sensing element with a physicochemical transducer, which aids in the detection of specific substances. The combination of biosensors with artificial intelligence (AI), machine learning (ML), and deep learning (DL) models can help distinguish true signals from false signals (noise), thereby enhancing the accuracy of the biosensor and enabling the data processing necessary to extract meaningful information [127].

Apps like PestID and PestNet, made by the University of Georgia and the Food and Agriculture Organization (FAO, respectively, allows its users to detect and report plant diseases through the use of the smartphone camera, and comparing the images to database knowledge and experts opinions, offering ways for pest control methods and management strategies causing for an isolated and controlled use of pesticides [124].

These systems can identify changes in agricultural produce, both in the fields and during post-harvest storage, through the use of sensors and radars, analyzing surface and chemical/spectral characteristics in real-time, enabling the detection of high-risk products before they are processed and distributed, thereby contributing to food safety [128].

The use of AI-derived models for pesticide analyses has multiple advantages, form being a quick and precise alternative due to the data evaluation capacity, to being a non-invasive and non-destructive approach, and something that can be easily incorporated in the existing equipment [116].

The widespread implementation is restricted due to a lack of data, particularly for uncommon pollutants or small amounts of residues, and because of challenges in combining hardware, since high-quality hyperspectral systems are still costly. Artificial intelligence models need to make decisions that can be explained, as regulatory bodies are more frequently asking for clear insights into models for non-damaging testing methods used in the industry [129].

### 8.4. Consumer-Level Guidance and Smart Processing Recommendations

AI is also beginning to influence consumer-level decisions, especially in the fields of food safety, food quality, and nutritional content. The creation of apps utilizing AI, deep learning, and advanced imaging and spectroscopic techniques can help consumers gain insight into the pesticide residues present in produce and identify the best methods for their mitigation [130]. With the integration of AI-powered vision, the systems can identify anomalies by comparing visual attributes with specified standards, thereby finding flaws, especially those related to pesticide contamination [131].

With more importance being given to the nutritional value of food, due to individual health concerns, apps powered by AI can be helpful to provide the optimal ways of food processing to achieve the most nutritional composition, as well as to create a personalized guide for food handling and preparation based on individual preference [130,131].

The incorporation of AI-powered chatbots in the food industry, particularly in the restaurant sector, enables consumers to receive a personalized experience that promotes healthier choices, taking into account nutritional value and food quality, while meeting the needs and preferences of targeted customers [132].

These innovations empower consumers to make informed decisions that reduce dietary pesticide intake.

Challenges consist of differences in data (such as lighting, camera quality, and handling situations), misunderstandings by users of AI results, and worries about the truthfulness of food safety statements from consumer applications. In order to build trust, models need to be clear and offer estimates of uncertainty instead of absolute assertions [129].

### 8.5. Regulatory Analytics and Policy Development

On a broader scale, AI is being employed to analyze large databases of pesticide residue monitoring data (e.g., from EFSA, FDA). Regulatory agencies utilize AI in the processing of large amounts of data in a short time, as well as in the development of new methods for risk assessment [133]. These models enable regulatory agencies to identify and track trends in non-compliance, predict high-risk commodities or regions using real-time data management, and refine sampling strategies and inspection frequencies [134].

With constant access to data and reliance on the various models provided by AI, we can support data-driven policy and ensure effective monitoring and risk management across the supply chain [135].

Although AI does not physically remove pesticide residues, it plays an essential indirect role in their mitigation by enhancing prediction, prevention, and control strategies. Its integration into agricultural practices, processing technologies, and regulatory systems allows for a more proactive and data-driven approach to food safety. As AI technologies continue to evolve, their contribution to minimizing pesticide residues and ensuring safe, sustainable food production will become even more significant.

Regulatory approval is still restricted since a lot of AI systems do not have clarity, set validation procedures, and defined error limits. It is important to guarantee that AI can be checked, tracked, and easily explained in order to incorporate it into formal risk evaluation methods [136].

### 8.6. Key Challenges in Implementing AI Systems

Globally, the use of AI systems for pesticide control, aiming to ensure food safety and support necessary regulations, presents some difficulties, such as a lack of data, the clarity of the models, and acceptance by regulatory bodies. The limited availability of data remains a major problem, since collecting high-quality information on pesticide performance, new contaminants, changes in different environments, and long-term observations is uncommon, expensive, and generally non-uniform. This scarcity of adequate data limits the robustness of the models and hinders their large-scale application across diverse crops, climates, cultivation methods, and locations [137]. 

The clarity of the model is also a very important topic. In areas such as agriculture and food security, where choices impact human health, environmental care, and compliance with regulations, stakeholders need to trust, understand, and explain the results of the algorithms. Methods for explainable artificial intelligence, such as SHAP, LIME, Grad-CAM, and transparent boosting models, offer ways to improve understanding, but their use is not yet common and can overload computers [138]. 

These limitations converge in the challenge of regulatory acceptance. Agencies responsible for pesticide approval, food-safety oversight, and public-health protection require transparent, validated, and reproducible models with well-defined uncertainty estimates. The absence of harmonized standards for evaluating AI systems slows formal integration, leaving conventional empirical and laboratory-based approaches as the default basis for regulatory decision-making.

Regarding into the future, different improvements in technology and methods present encouraging options to tackle these limitations. Digital Twins (DTs)powered by AI can combine live data from sensors, environmental checks, and chemical behavior models to mimic how crops grow, how pesticides break down, and how supply chains operate. These systems can help in testing different scenarios, assessing risks, and managing changes [139]. Models that blend mechanistic and AI approaches, which incorporate scientific and physical knowledge into data-centered designs, might enhance understanding and better match what regulations require [140]. 

## 9. Conclusions and Future Perspectives

Minimizing pesticide residues in food relies on both effective decontamination techniques and reliable analytical monitoring. At the household level, simple practices such as washing with mild chemical solutions, peeling, and heat treatments like boiling or blanching remain the most practical and effective. In industrial settings, advanced methods—including ozonated water, cold plasma, and ultrasound-assisted washing—have demonstrated strong efficiency while preserving organoleptic properties. Furthermore, microbial fermentation and photocatalysis have shown promising results, particularly in targeting specific pesticide classes.

Pesticide residue quantification must be carried out using a validated analytical method that is aligned with international standards (e.g., SANTE/11312/2021 [31], Codex Alimentarius, EPA protocols). Generating reliable data is crucial not only for evaluating the effectiveness of decontamination strategies but also for meeting regulatory requirements and ensuring consumer health safety.

Commonly investigated pesticide models include chlorpyrifos (an organophosphate), cypermethrin and deltamethrin (pyrethroids), imidacloprid (a neonicotinoid), and carbendazim and mancozeb (fungicides). These are typically chosen because of their high prevalence, environmental persistence, and significant toxicological profiles.

Looking ahead, a major development in pesticide residue management is the growing use of Artificial Intelligence (AI). New AI-based tools are being designed to estimate residue levels at harvest by combining information on weather patterns, crop characteristics, and application practices. These systems can also support the selection of effective decontamination strategies through machine-learning models and deliver real-time recommendations to regulators and farmers to help prevent unnecessary pesticide use. Progress in analytical methods, improved processing technologies, and AI-supported decision platforms together offer a promising route to more sustainable food safety and reduced consumer exposure.

Strengthening expertise in AI will further enhance decision-making directly in the field and during production, enabling fast on-site analyses without relying on cloud computing. Equally important is the creation of large, openly accessible, and standardized datasets that capture a wide range of agricultural environments, contaminant profiles, and residue behaviors—an essential step for building robust, globally applicable models. Establishing shared principles for validating and interpreting AI systems, along with clear performance benchmarks, will also be necessary to ensure their safe and dependable use.

## Figures and Tables

**Table 1:** Key Findings from EFSA’s 2021, 2022, and 2023 reports.

Report	EFSA 2021 Report [8]		EFSA 2022 Report [9]		EFSA 2023 Report [10]	
Date of Publication	26th April 2023		23rd May 2024		14th May 2025	
Control Program	National control programme	EU-coordinated control program	National control programme	EU-coordinated control program	National control programme	EU-coordinated control program
Number of samples	87.863	13.845	110.829	11.727	132.793	13.246
Compliant	96.1%	98.7%	96.3%	99.1%	98%	99%
Non-Compliant	3.9%	1.3%	3.7%	0.9%	2%	1%

**Table 2:** Summary of the effects of washing on the reduction of pesticide residues.

Food Material	Pesticide	Processing Conditions	Percentage of Reduction	Reference
Lettuce	Chlorfenapyr	Rinsed under running tap water for 5 min	82.5 ± 1.3	[78]
Perilla Leaves	Pyraclostrobin		76.3 ± 4.5	
Spinach	Chlorantraniliprole		87.2 ± 2.1	
Crown Daisy	Azoxystrobin		78.5 ± 0.8	
Ssamchoo	Fludioxonil		59.7 ± 4.1	
	Cadusafos	Washed with mineral water and soaked for 20 min	65.8	[79]
Long-grain Rice	Fenamidone		65.9	
	Pirimiphos-methyl		73.0	
	Bupirimate		40.7	
Brown Rice	Carbaryl		56.8	
	Fenpropidin		47.8	
	Ethoprophos		63.5	
Basmati Rice	SpinosadA		51.3	
	Tetraconazole		53.1	
Tomato	Difenoconazole metabolite CGA205375	Washed with tap water for 5 min	82.05	[80]
Lettuce	Thiamethoxam	Soaked in water with vegetable detergent for 5 min	57.5 ± 2.3	[78]
Perilla Leaves	Indoxacarb		45.8 ± 5.1	
Spinach	Lufenuron		43.9 ± 8.3	
Crown Daisy	Diniconazole		38.6 ± 4.5	
Ssamchoo	Imidacloprid		32.6 ± 2.7	
	Bupirimate	Washed with mineral water and soaked in water and vinegar for 20 min	67.1	[79]
Long-grain Rice	Difenoconazole		68.4	
	Spiroxamine		76.6	
	Cadusafos		69.7	
Brown Rice	Fenthion sulfoxide		65.8	
	Phoxim		70.2	
	Prothioconazole desthio		58.6	
Basmati Rice	Fenamidone		60.6	
	Azoxystrobin		50.4	

**Table 3:** Summary of the effects of blanching on the reduction in pesticide residues.

Food Material	Pesticide	Processing Conditions	Percentage of Reduction	Reference
Spinach	Propamocarb	2 min at 80–100 °C	41.0	[96]
	Chlorantraniliprole		30.0	
Cowpeas	Cyromazine		67.6	[97]
	Acetamiprid		60.9	
Chinese Kale	Carbofuran		69.0	[98]
	Deltamethrin		45.0	
Yard Long Bean	Captan		80.0	
	Flumethrin		59.0	

**Table 4:** Comparison of the advantages and limitations of innovative methods to mitigate the level of pesticide residues in samples.

Method	Principle	Advantages	Limitations	References
Ozonated Water Washing	Ozone (O_3_) is a powerful oxidizing agent that breaks down pesticide molecules into less toxic or inert compounds	Leaves no chemical residues and also disinfects microbial contaminants	Ozone is short-lived in solution and must be continuously generated High concentrations may damage produce quality (discoloration, off-flavors) Careful control needed to avoid worker respiratory exposure	[103]
Ultrasonification	High-frequency sound waves generate cavitation bubbles in water, which collapses and create localized heat and turbulence, which disrupts surface pesticide films	Eco-friendly, non thermal, improves cleaning without damaging texture	Efficacy depends on duration, ultrasonic frequency, and pesticide type Equipment cost and scale-up in domestic settings is a barrier	[104]
Electrolyzed Water Devices (EWDs)	Generate electrolyzed water solutions (acidic, neutral, or alkaline) by passing an electric current through water containing a small amount of salt. The process produces: Hypochlorous acid (HOCl) and free chlorine (in acidic electrolyzed water, AEW); Sodium hydroxide (NaOH) (in alkaline electrolyzed water, AIEW)	Effective residue removal Non-toxic Quick and scalable Also disinfects	Limited penetration Sensitive to pH and temperature Equipment must be corrosion-resistant due to free chlorine Not effective for all pesticides classes, especially highly lipophilic or systemic compounds	[105]
Cold Plasma	Plasma (ionized gas) produces reactive oxygen and nitrogen species that degrade pesticide residue on food surfaces	Non-thermal, minimal quality impact, and high removal efficiency	Some pesticides (e.g., chlorpyrifos) show resistance under typical plasma conditions Potential formation of unknown degradation by products: safety and regulatory approval remain challenging Scaling up and process standardization is still under development	[99]
Enzymatic Degradation	Use of specific enzymes (e.g., organophosphorus hydrolases, laccases) that target and degrade pesticide molecule	High specificity: enzymes like organophosphorus hydrolases can break down particular pesticide classes Biodegradable process producing minimal toxicity	Enzyme production is costly; activity can be sensitive to pH and temperature Delivery in practical food processing systems remains under exploration	[106]
Photocatalysis (TiO_2_-UV Treatment)	Photocatalytic materials like titanium dioxide (TiO_2_) generate reactive radicals under UV light, degrading pesticides molecules on the surface	Can be implemented in washing units or grain surface treatment	UV exposure may damage some foods or degrade nutrients Only surface residues are affected, penetration in negligible	[107]
Edible Coatings with Detoxifying Agents	Active edible coatings (e.g., made from chitosan, alginate) enriched with detoxifying agents like activated carbon, plant extracts, or enzymes	Active coatings can absorb or degrade residual pesticides during storage Can concurrently extend shelf-life and reduce residue exposure	Effectiveness depends on coating uniformity and interaction time Regulatory hurdles for introduction of new coating formulas	[108]
Microbial Bioremediation	Use of non-pathogenic bacteria or fungi capable of metabolizing or biding pesticides residues	Certain non pathogenic bacteria or fungal species can metabolize or bind pesticide molecules into inert forms Potentially scalable in fermentative or wash treatments	Specificity to pesticide types, potential biocontrol or spoilage risk if not carefully selected Regulatory and safety evaluations needed for live microbial agents Mostly applied to environmental remediation, limited food use	[109]

## Data Availability

No new data were created or analyzed in this study. Data sharing is not applicable to this article.

## Abbreviations

The following abbreviations are used in this manuscript:

3-CA	3-Chloroaniline
AI	Artificial Intelligence
ANN	Artificial Neural Networks
BHC	Benzenehexachloride
CAC	Codex Alimentarius Commission
CNN	Convolutional Neural Networks
DDT	Dichlorodiphenyltrichloroethane
DL	Deep Learning
DOMs	Degrees of Milling
DTs	Digital Twins
EFSA	European Food Safety Authority
EPA	United States Environmental Protection Agency
EU	European Union
EWDs	Electrolyzed Water Devices
FAO	Food and Agriculture Organization of the United Nations
GAP	Good Agricultural Practices
GC-MS/MS	Gas Chromatography coupled with tandem Mass Spectrometry
IPM	Integrated Pest Management
IRAC	Insecticide Resistance Action Committee
JMRP	Joint FAO/WHO Meeting on Pesticide Residues
LAB	Lactic Acid Bacteria
LC-MS/MS	Liquid Chromatography coupled with tandem Mass Spectrometry
Log Kow	Octanol-Water Partition Coefficient
LOQ	Limit of Quantification
ML	Machine Learning
MRLs	Maximum Residue Limits
NIPH	Mexican National Institute of Public Health
OD600	Optical Density at 600 nm
PAN	Pesticide Action Network
PFAS	Per- and polyfluoroalkyl subs
PFs	Processing Factors
PHI	Pre-harvest Interval
PRIMo	Pesticide Residues Intake Model
PVPP	Polyvinylpolypyrrolidone
SUD	Sustainable Use of pesticides Directive
SVM	Support Vector Machine
UAVs	Unmanned Aerial Vehicles
UV	Ultraviolet
WHO	World Health
